# Prostate Cancer Characteristics Associated with Response to Pre-Receptor Targeting of the Androgen Axis

**DOI:** 10.1371/journal.pone.0111545

**Published:** 2014-10-30

**Authors:** Elahe A. Mostaghel, Andrew Morgan, Xiaotun Zhang, Brett T. Marck, Jing Xia, Rachel Hunter-Merrill, Roman Gulati, Stephen Plymate, Robert L. Vessella, Eva Corey, Celestia S. Higano, Alvin M. Matsumoto, R. Bruce Montgomery, Peter S. Nelson

**Affiliations:** 1 Division of Clinical Research, Fred Hutchinson Cancer Research Center, Seattle, Washington, United States of America; 2 Department of Medicine, University of Washington School of Medicine, Seattle, Washington, United States of America; 3 Department of Urology, University of Washington School of Medicine, Seattle, Washington, United States of America; 4 Geriatric Research, Education and Clinical Center, VA Puget Sound Health Care System, Seattle, Washington, United States of America; 5 Division of Public Health Sciences, Fred Hutchinson Cancer Research Center, Seattle, Washington, United States of America; UC Davis Comprehensive Cancer Center, United States of America

## Abstract

**Background:**

Factors influencing differential responses of prostate tumors to androgen receptor (AR) axis-directed therapeutics are poorly understood, and predictors of treatment efficacy are needed. We hypothesized that the efficacy of inhibiting DHT ligand synthesis would associate with intra-tumoral androgen ratios indicative of relative dependence on DHT-mediated growth.

**Methods:**

We characterized two androgen-sensitive prostate cancer xenograft models after androgen suppression by castration in combination with the SRD5A inhibitor, dutasteride, as well as a panel of castration resistant metastases obtained via rapid autopsy.

**Results:**

In LuCaP35 tumors (intra-tumoral T:DHT ratio 2∶1) dutasteride suppressed DHT to 0.02 ng/gm and prolonged survival vs. castration alone (337 vs.152 days, HR 2.8, p = 0.0015). In LuCaP96 tumors (T:DHT 10∶1), survival was not improved despite similar DHT reduction (0.02 ng/gm). LuCaP35 demonstrated higher expression of steroid biosynthetic enzymes maintaining DHT levels (5-fold higher SRD5A1, 41 fold higher, 99-fold higher RL-HSD, p<0.0001 for both), reconstitution of intra-tumoral DHT (to ∼30% of untreated tumors), and ∼2 fold increased expression of full length AR. In contrast, LuCaP96 demonstrated higher levels of steroid catabolizing enzymes (6.9-fold higher AKR1C2, 3000-fold higher UGT2B15, p = 0.002 and p<0.0001 respectively), persistent suppression of intra-tumoral DHT, and 6–8 fold induction of full length AR and the ligand independent V7 AR splice variant. Human metastases demonstrated bio-active androgen levels and AR full length and AR splice-variant expression consistent with the range observed in xenografts.

**Conclusions:**

Intrinsic differences in basal steroidogenesis, as well as variable expression of full length and splice-variant AR, associate with response and resistance to pre-receptor AR ligand suppression. Expression of steroidogenic enzymes and AR isoforms may serve as potential biomarkers of sensitivity to potent AR-axis inhibition and should be validated in additional models.

## Introduction

Although initially highly effective, androgen deprivation therapy for prostate cancer is uniformly marked by progression to castration resistant prostate cancer (CRPC) over a period of 18–36 months, with an ensuing median survival of 1–2 years. [1] While androgen receptor (AR)-regulated gene expression is initially suppressed, [2], [3] the transcriptional program regulated by the AR is reactivated in CRPC via mechanisms that maintain both ligand and receptor mediated contributions to AR signaling. [4]–[6] These include residual intratumoral androgens at levels sufficient to activate the AR program, [7]–[9] and increased AR activity, either via amplification or overexpression (resulting in enhanced sensitivity to low ligand levels) [10], [11] or via the recently described expression of truncated ligand-independent AR splice variants (AR^sv^) lacking the ligand binding domain (LBD). [12]–[17] Notably, studies of maximal or combined androgen blockade utilizing steroidal or non-steroidal AR antagonists that target the AR LBD have only demonstrated a small, albeit statistically significant, improvement in 5 year survival rates (27.6 vs. 24.7%; p = 0.005). [18] The most straightforward explanation for the lack of greater efficacy is the inability of these AR antagonists to outcompete residual testosterone (T) and dihydrotestosterone (DHT) (which have AR binding affinities that far exceed those of synthetic anti-androgens), [19] or a failure to effectively target AR^sv^ lacking the LBD.

The clinical responses to new potent AR antagonists and novel steroid synthesis inhibitors, coupled with evidence of intracrine androgen biosynthesis in prostate cancer suggest that concepts of maximal androgen blockade should be revisited and refined. A recent study designed to target multiple steps in androgen biosynthesis in men failing ADT (using hydrocortisone, the CYP17A1 inhibitor ketoconazole, and the steroid 5-alpha-reductase inhibitor (SRD5A) dutasteride) produced substantial PSA declines in most patients and a median response duration of 20 months. [20] Thus, clinical efficacy may not require an AR antagonist in the setting of more effective suppression of ‘pre-receptor’ signaling through reduction of AR ligands. Importantly, the effectiveness of inhibiting AR activation in prostate cancers, either through enhanced pre-receptor approaches or those directly targeting AR, varies substantially between patients, and the factors contributing to these differential outcomes are unknown. For example, prevention trials using SRD5A inhibitors significantly decreased the rate of detecting localized prostate cancers, but this intervention was clearly not effective in preventing cancer progression or detection in all men. [21], [22] Similarly, recent clinical trials of new inhibitors of CYP17A1 and AR in men with CRPC have reported substantial inter-patient differences in objective responses [23], [24].

In this study, we sought to determine tumor-specific characteristics that influence response of prostate cancers to inhibition of pre-receptor AR signaling, using androgen deprivation combined with the dual SRD5A inhibitor dutasteride. T and DHT are biologically active high-affinity AR ligands. However, between 3–10 fold higher concentrations of T are required to exert the same AR-mediated transcriptional effects of DHT. [25], [26] Thus, reducing DHT levels by inhibiting conversion of T to DHT may represent an effective pre-receptor method to suppress the overall magnitude of bioactive AR signaling. We hypothesized that the impact of SRD5A inhibition on tumor growth would associate with intra-tumoral androgen levels and/or the expression of steroid biosynthetic enzymes suggestive of a relative dependence on DHT-mediated growth. Factors determining the relative response of prostate tumors to pre-receptor androgen suppression with agents such as SRD5A inhibitors are unknown, but could serve as predictors of efficacy for these agents in prostate cancer prevention, or as a component of ADT in advanced disease.

## Materials and Methods

### LuCaP Human Prostate Cancer Xenografts

This study was carried out in strict accordance with the recommendations in the Guide for the Care and Use of Laboratory Animals of the National Institutes of Health. All experiments involving animals were performed in accordance with protocols approved by the Fred Hutchinson Center Institutional Animal Care Use Committee (file 1775). All surgery was performed under isofluorane anesthesia, and all efforts were made to minimize suffering. The LuCaP35 and LuCaP96 lines are patient derived xenografts that were established as part of the University of Washington tissue bank as previously described [27], [28] and were selected for analysis as both lines demonstrate castration sensitive growth in intact male mice. Briefly, LuCaP35 was derived from a lymph node metastasis obtained from a 66-year old Caucasian male with CRPC. LuCaP96 was derived from transurethral resection of a primary Gleason 9 prostate carcinoma obtained in a 61 year-old Caucasian male one month prior to documentation of castration-resistant disease. In both xenografts the AR exonic DNA sequence is wild-type (data not shown).

Male C.B-17 SCID mice (Charles River Laboratories, Wilmington MA) were implanted subcutaneously with 30 mm^3^ tumor pieces. When tumors reached an average of 300 mm^3^, mice (LuCaP35 n = 62; LuCaP96 n = 65) were castrated (Cx) and randomized to treatment with vehicle or dutasteride (Dut) therapy over 8 weeks. Dutasteride was administered (PEG monolaurate (Sigma 460133)/1% Tween 80 (Sigma P4780)) by oral gavage 5 days per week in a volume of 200µl. Tumor volume was determined by the following formula (long and short axis lengths in mm): long×(Short∧2)/2. Tumors from a subset of mice in each cohort were harvested at early time points of treatment (tumor size of ∼500 mm^3^; range 7–21 days). When tumors reached approximately 750–1000 mg in size, the animals were euthanized according to institutional protocol and the xenografts harvested and flash frozen for determination of tissue androgens and extraction of total RNA. The average number of days mice in the Cx+Dut groups had been off dutasteride treatment at the time tumors were resected was 211 days for LuCaP35, and 96 days for LuCaP96. Dutasteride was generously provided by GlaxoSmithKline.

All procedures involving human subjects were approved by the Institutional Review Board of the University of Washington Medical Center, and all subjects signed written informed consent. Patients with metastatic prostate cancer underwent rapid autopsies under the aegis of the University of Washington Prostate Cancer Donor Autopsy Program as previously described [9], [27]. Autopsies were performed within 4–10 hours of death. Samples of all gross metastatic tumor sites were obtained under sterile conditions and the site and volume of osseous and non-osseous metastases were recorded. Fresh tissue was snap frozen in liquid nitrogen immediately after harvesting and maintained at −80°C.

### Steroid Measurements

Androgen levels in xenograft tissues were determined by mass spectrometry (MS) using methods we have described. [29] This procedure resulted in a lower limit of quantitation of 1 pg per sample for testosterone and DHT. Intra-assay coefficients of variation generated using human serum for high, mid and low-range samples were 3.5, 3.1 and 3.8% for testosterone and 6.3, 4.3 and 15.8% for DHT respectively.

### RNA Isolation, quantitative RT-PCR and immunohistochemistry

Samples were used for RNA isolation, cDNA synthesis and quantitative RT-PCR using methods and primers we have previously described. [9], [30] Immunohistochemical staining and scoring for AR and PSA was carried out as we have previously described, [31] using a polyclonal anti-PSA antibody (DAKO, Inc.), an anti-AR antibody directed at the N terminus (clone F36.4.1, Biogenex), and an anti-AR antibody directed at the C terminus (C-19, Santa Cruz). Negative control immunostains, substituting preimmune immunoglobulin of the same species as that in which the antibody was generated, showed no reaction product.

### Statistical Analyses

To evaluate differences in tumor volume trajectory, we first determined change points in the tumor growth curves using t-tests to sequentially compare tumor volumes every two days. After segmenting the curves, we applied a linear mixed model to quantify the treatment effect on tumor growth for each linear period by regressing log tumor volume on day of measurement, on treatment group, and on an interaction between day and treatment while accounting for variability in initial tumor volume across mice. Progression free survival in castration vs. castration + dutasteride treated mice (defined as tumor size <750 mm^3^) was determined via Kaplan Meier analysis with comparison of curves using the Mantel-Haenszel logrank test. To predict the approximate sample size in each treatment group that might be necessary to detect a statistically significant effect of castration plus dutasteride versus castration alone, we utilized complete case resampling for a range of sample sizes n = 10, 20, …, 200 over 500 bootstrap replicates. For each sample size and bootstrap replicate, we fit a Cox proportional hazards model and recorded the estimated hazard ratio and p-value.

For analysis of qRT-PCR data, the mean cycle threshold (Ct) for each gene was normalized to expression of the housekeeping gene RPL13A in the same sample (delta Ct). The mean expression of RPL13A was 15.6+/−0.5 (mean +/− SD) and 14.1+/−0.8 in LuCaP35 and LuCap96 tumors respectively. Welch’s two sample t-tests were used to compare differences in androgen levels and differences in mean delta Ct’s for each gene between treatment groups without correction for multiple testing. P values<0.05 were considered significant. The fold change was calculated by the delta-delta Ct method (fold = 2^ΔΔCt^). The raw dCT’s, ddCT’s and fold changes are presented in the Supplemental data.

## Results

### Prostate cancers responding to androgen suppression demonstrate intrinsic differences in basal levels of intratumoral androgens and steroidogenic gene expression

**Figure 1:**
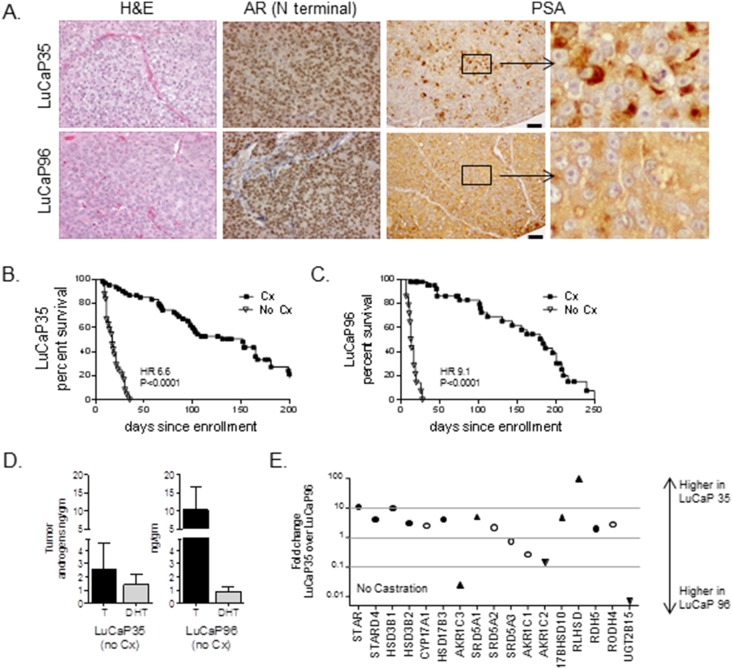
Characterization of LuCaP35 and LuCaP96 prostate cancer xenografts and responses to systemic androgen suppression. (**A**) Representative FFPE samples of each xenograft were stained with hematoxylin and eosin (H&E) and for expression of the androgen receptor (AR) and PSA as indicated. The scale bar (depicted on the PSA figures for ease of visualization) are 50µm. Kaplan-Meier plots of progression free survival (defined as tumor size <750 mm^3^) in mice bearing LuCaP35 (**B**) or LuCaP96 (**C**) xenografts. Intact male SCID mice were implanted subcutaneously with 30 mm^3^ pieces of the indicated xenografts. When tumors reached ∼300 mm^3^, mice were randomly enrolled into cohorts that were either left intact (No Cx) or castrated (Cx). P-values for curve comparisons were generated using the Mantel-Haenszel logrank test. (**D**) Mean and standard deviation of tissue testosterone (T, black bar) and DHT (gray bar) levels measured by mass spectrometry in tumors of the indicated xenograft (passaged in intact mice). (**E**) Relative expression of transcripts for the indicated steroidogenic genes was calculated using the delta dCt method (fold change = 2∧ddCt). Genes differentially expressed in LuCaP35 vs. LuCaP96 within one order of magnitude are indicated within the gray lines. Significant differences (by Welch’s t test; p<0.05) are indicated by black circles; white circles indicate genes that were not significantly different between LuCaP35 and LuCaP96 (all values given in Supplementary Data 2).Upward triangles indicate highly differentially expressed genes specifically leading to increased T (AKR1C3, 40 fold) or increased DHT levels (SRD5A1, 5.0 fold; 17BHSD10 4.8 fold; RLHSD, 99 fold). Downward triangles indicate highly differentially expressed genes specifically mediating DHT catabolism (AKR1C2, 7 fold; UGT2B15, 3000 fold).

To evaluate tumor characteristics associated with response and resistance to AR pathway-directed therapy, we first determined intratumoral androgens and steroidogenic gene expression in two prostate cancer patient derived xenografts, LuCaP35 and LuCaP96. These lines express AR and PSA, (**Figure 1A**) and respond to castration with tumor regression and prolongation in progression-free survival (PFS), but ultimately progress to castration-resistant growth (p<0.0001 for both; **Figure 1B, 1C**). Notably, LuCaP35 and LuCaP96 tumors resected from mice with intact gonadal function exhibited marked differences in basal levels of intratumoral androgens (**Figure 1D**): LuCaP96 tumors have higher levels of T and similar levels of DHT, and thus a higher ratio of intratumoral T:DHT compared to LuCaP35 tumors, at 10∶1 and 2∶1, respectively (LuCaP96 T = 10.2±6.5 ng/gm; DHT = 0.9±0.4 ng/gm; LuCaP35 T = 2.6±2.0 ng/gm; DHT = 1.4±0.8 ng/gm, **Table S1**).

To identify mechanisms contributing to basal differences in intra-tumoral T and DHT concentrations, we evaluated tumor-specific expression of transcripts encoding the primary enzymes involved in steroid synthesis and degradation (**Figure S1**). While many steroidogenic genes were expressed in LuCaP35 within one order of magnitude of their expression in LuCaP96 (**Figure 1E**), several differences consistent with the different tumor androgen profiles were present. In particular, gene expression in LuCaP35 was consistent with androgen production and maintenance of DHT levels, demonstrating higher levels of steroid biosynthetic genes such as STAR (10-fold) and HSD3B1 (10-fold), higher expression of genes mediating DHT production (SRD5A1, 5-fold; 17BHSD10 4.8-fold, RLHSD, 99-fold), and lower expression of genes mediating DHT catabolism (AKR1C2, 7-fold lower; UGT2B17, 3000-fold lower). In contrast, gene expression in LuCaP96 was consistent with production and maintenance of T, with significantly higher levels of AKR1C3 (40-fold; consistent with an increase in T production from androstenedione), in conjunction with lower levels of SRD5A1 (the primary enzyme responsible for conversion of T to DHT in neoplastic prostate tissue) [32] and higher levels of the DHT catabolism enzymes AKR1C2 and UGT2B17. (Cycle thresholds and fold changes for all genes in Figure 1E are presented in **Table S2**). Since these tumors were propagated in genetically-identical murine hosts, these data identify intrinsic tumor-specific variation in androgen metabolic programs culminating in marked intratumoral differences in AR ligand concentrations.

### Pre-receptor androgen pathway suppression with castration plus SRD5A inhibition demonstrates a tumor-specific impact in delaying progression to CRPC

Given the 5–10 fold higher potency of DHT in engaging and activating AR compared to T, [25], [26] strategies targeting DHT reduction by inhibiting metabolism of T to DHT may represent an effective pre-receptor method to suppress the overall magnitude of AR signaling. To determine whether tumor-specific differences in the basal ratio of intratumoral T:DHT would influence clinical response to agents targeting conversion of T to DHT, we treated cohorts of mice bearing LuCaP35 and LuCaP96 tumors with castration alone, or castration plus the dual SRD5A inhibitor dutasteride. Intact male SCID mice were subcutaneously implanted with 30 mm^3^ pieces of LuCaP35 or LuCaP96 tumors. After growth to ∼300 mm^3^, mice were castrated and randomized to vehicle or dutasteride administered by oral gavage for 8 weeks.

**Figure 2:**
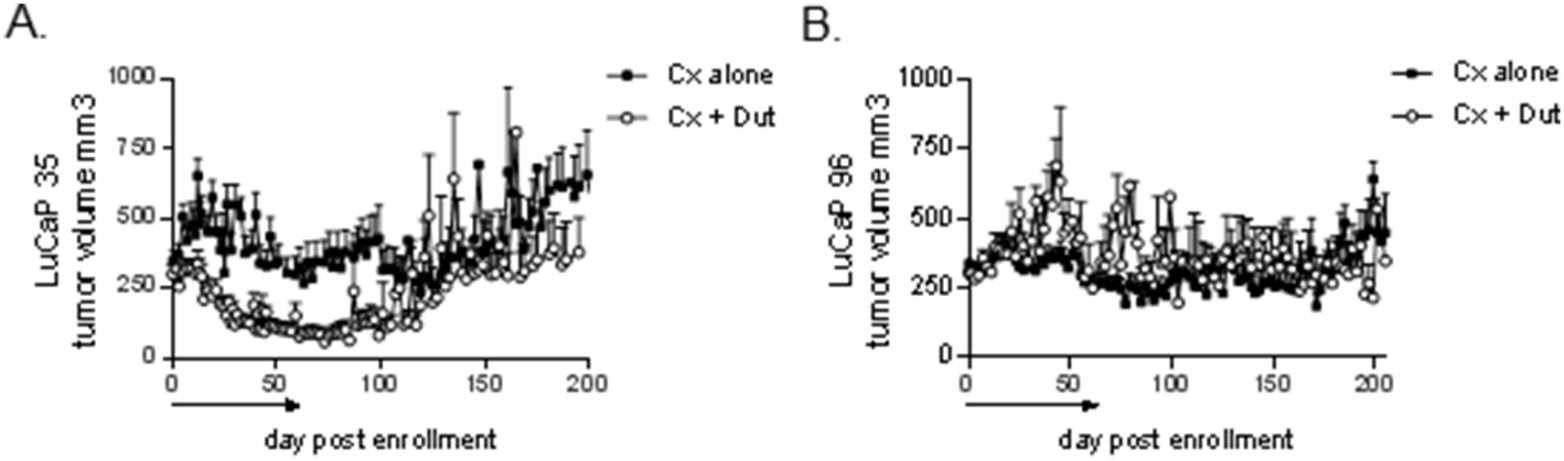
Tumor growth and androgen levels in prostate cancer xenografts treated with castration and dutasteride. Mean tumor volumes in mice bearing LuCaP35 (**A**) and LuCaP96 (**B**) xenografts. Intact male SCID mice were subcutaneously implanted with 30 mm^3^ pieces of the indicated xenograft. When tumors reached ∼300mm^3^, mice were castrated and randomly enrolled into cohorts treated with either vehicle (Cx) or dutasteride (Cx+Dut) for 8 weeks (denoted by black line above x-axis). Mean tumor volumes are depicted for each treatment group at the indicated days post enrollment (Cx, black squares; Cx+Dut, white circles).

Mean tumor volumes in LuCaP35 tumors were markedly suppressed by the combination of castration plus dutasteride vs. castration alone (**Figure 2A**). Tumor growth occurred in three distinct stages following initiation of treatment: a brief period of continued growth (days 0–14), a prolonged phase of tumor regression or stability (days 14–75), and a final phase of tumor re-growth (days 75–200). To better quantitate the impact of dutasteride, we compared tumor volume trajectories in castration vs. castration plus dutasteride groups using a linear mixed model applied to each post-treatment phase. Dutasteride plus castration significantly decreased the percent increase in tumor volume per day versus castration alone in the initial ‘on-treatment’ stage (from 3.2% [95%CI 2.7–3.7] to 0.9% [95%CI 0.2–2.0]; p<0.0001), and increased the percent of tumor regression per day in the subsequent phase (from 1.2% [95%CI 0.5–1.8] to 3.6% [95%CI 2.0–5.3]; p<0.0001). However, following discontinuation of therapy, dutasteride-treated tumors demonstrated more rapid percent re-growth per day (from 1.1% [95%CI 0.9–1.3] to 2.3% [95%CI 1.8–2.9]; p<0.0001), suggesting continued therapy would be required to maintain therapeutic efficacy.

In contrast, tumor volumes in LuCaP96 tumors were not suppressed by addition of dutasteride to castration (**Figure 2B**), with no change in the hazard ratio (HR) for PFS (HR 0.9, p = 0.97; not shown). To examine how much the non-significant difference was due to the number of animals, we bootstrapped from the original data 500 times and found that even with n = 170 mice per treatment arm, a modest (HR 1.4) but statistically significant improvement in survival was observed in only 50% of iterations, suggesting the likelihood of detecting a clinically meaningful treatment effect in a larger study was low. Overall, these observations demonstrate that pre-receptor androgen pathway suppression with castration plus SRD5A inhibition can delay progression to castration resistant growth. However this response is not universal, but rather appears to be tumor type-specific, and is associated with those tumors exhibiting an androgen metabolic enzyme profile directed at maintaining intratumoral DHT levels such as LuCaP35, rather than a T-predominant tumor such as LuCaP96.

### Tumor size at initiation of treatment influences the long-term response to androgen suppression plus SRD5A inhibition

**Figure 3:**
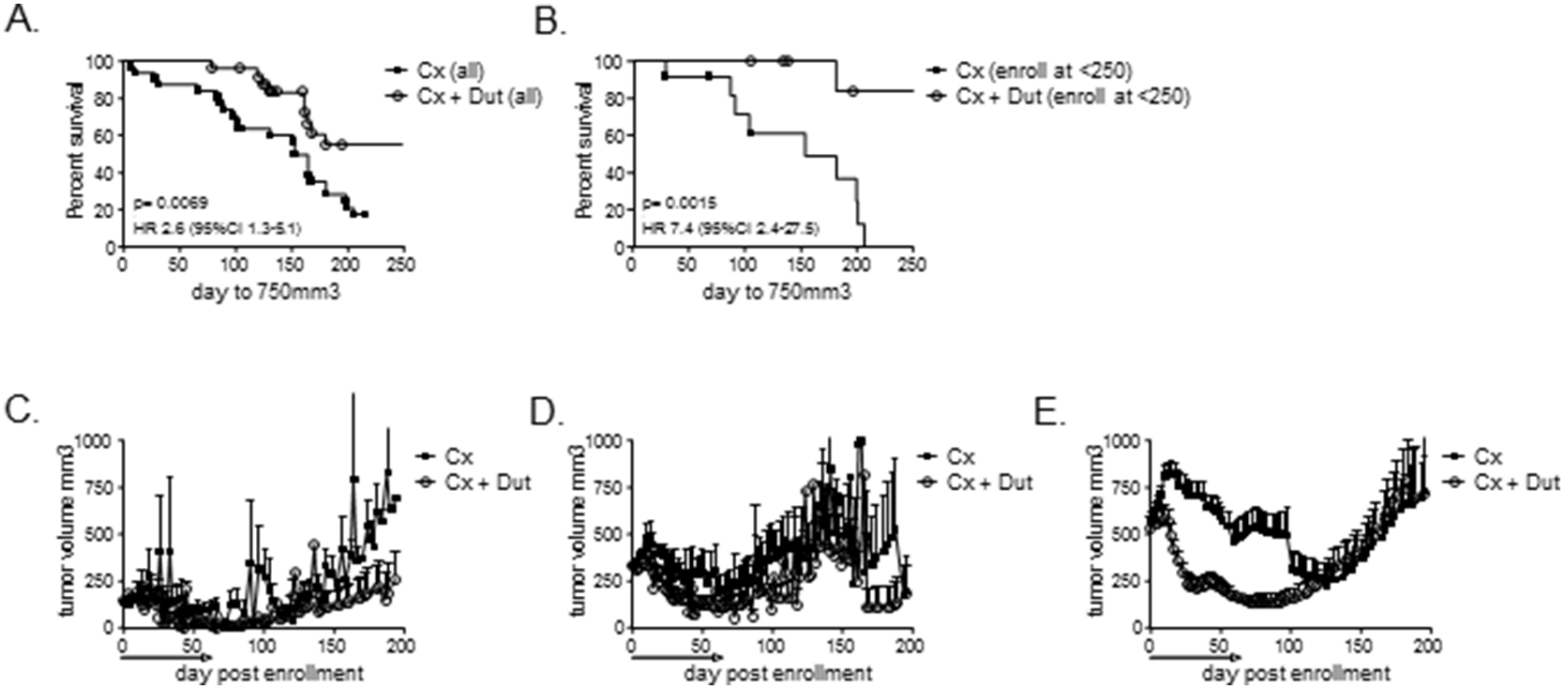
Response to dutasteride by tumor size at enrollment in LuCaP35 xenografts. Kaplan-Meier plots of progression free survival (defined as tumor size <750 mm3) in all LuCaP35 tumors treated with castration alone (Cx) vs. castration + dutasteride (Cx + Dut) (**A**), and in tumors enrolled into treatment when tumors were <250 mm^3^ (**B**). P-values for curve comparisons were generated using the Mantel-Haenszel logrank test. Mean tumor volume growth curves at the indicated days post enrollment in LuCaP35 tumors enrolled into treatment when tumors were <250 mm^3^ (**C**), between 250–400 mm^3^ (**D**), and >400 mm^3^ (**E**). Dutasteride treatment was continued for 8 weeks (denoted by black line above x-axis) in the castration + dutasteride group.

To further examine tumor characteristics associated with response to pre-receptor AR pathway inhibition, we determined whether response to treatment was related to tumor size at initiation of therapy. Although the study was designed to begin treatment at a tumor volume of ∼300 mm^3^, logistics of the animal experiments resulted in a measureable variation in tumor size at study entry. We arbitrarily grouped tumors into cohorts of <250 mm^3^, 250–400 mm^3^, and >400 mm^3^ at enrollment and compared the effect of castration vs. castration plus dutasteride within each group. Notably, the significant improvement in median survival imparted by dutasteride in the entire LuCaP35 cohort (from 152 to 337 days, HR for progression 2.8 [95% CI 1.4–4.6], p = 0.0015; **Figure 3A**), appeared predominately due to an impact on tumors that were smallest (<250 mm^3^) at the time of enrollment (HR for progression 7.3 [95%CI 2.2–30], p<0.0015, vs. castration alone; **Figure 3B**). In contrast, the combination of castration plus 8 weeks of dutasteride did not statistically impact PFS in tumors measuring 250–400 mm^3^ or >400 mm^3^ at enrollment (not shown); however, this may reflect decreased power associated with the post-hoc sub-classification of the groups.

Inspection of tumor volume curves suggested a suppressive effect of dutasteride was maintained even after discontinuation of therapy for tumors <250 mm^3^ at enrollment (**Figure 3C**). In contrast, tumor growth was suppressed relative to castration in tumors >250 mm^3^ at enrollment (**Figure 3D, 3E**), but only while tumors were under therapy. After discontinuation, tumor size in these cohorts caught up to tumors treated with castration alone, such that ultimately no difference in survival was observed. The starting tumor volume was not associated with survival in LuCaP96 tumors treated with dutasteride (not shown).

### Systemic androgen suppression plus SRD5A inhibition significantly decreases tumor DHT levels compared to androgen suppression alone

**Figure 4:**
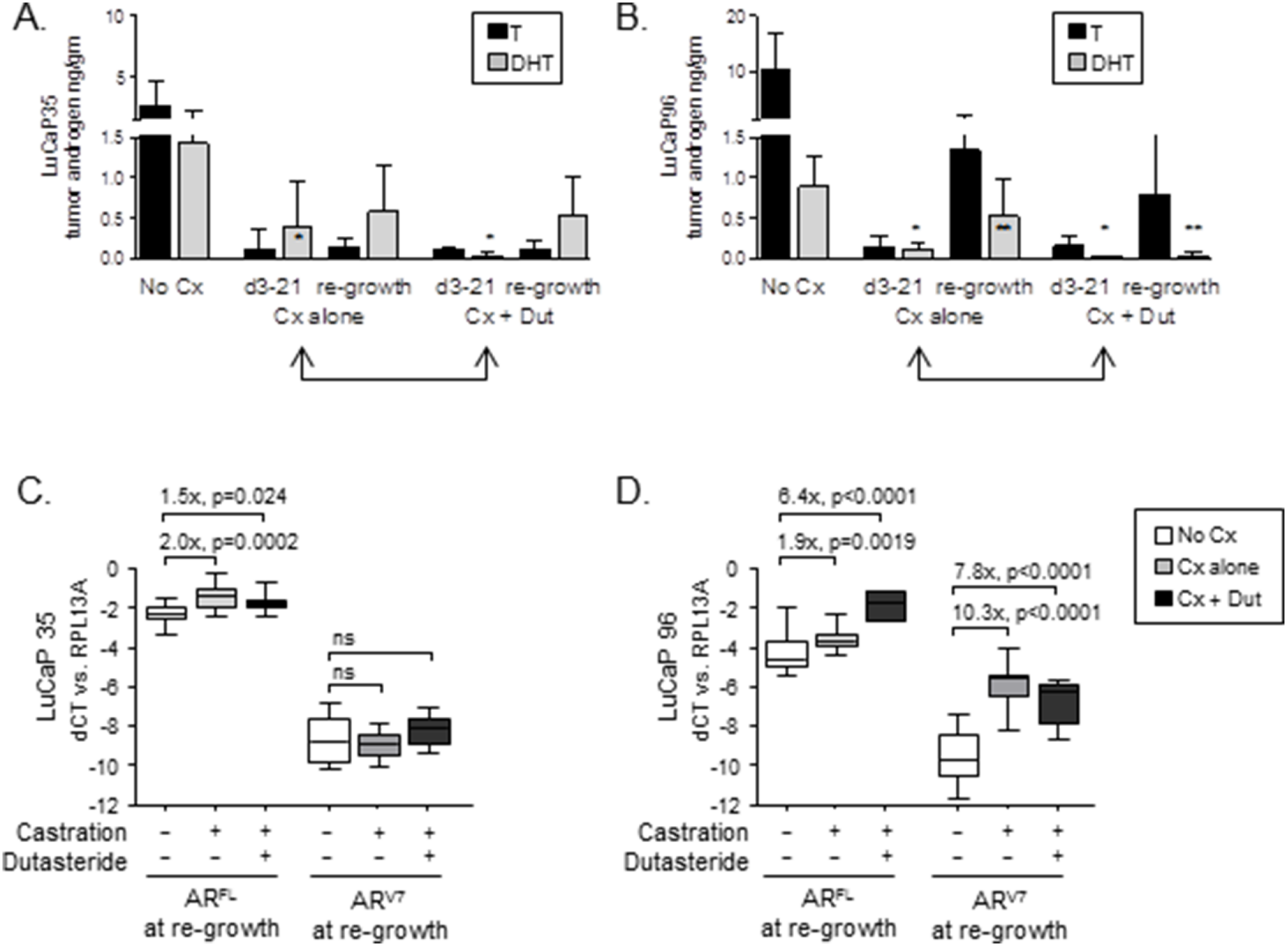
Androgen levels and AR expression in prostate cancer xenografts treated with castration and dutasteride. Tissue testosterone (T, black bars) and DHT (gray bars) levels were measured by mass spectrometry in LuCaP35 (**A**) and LuCap96 (**B**) tumors resected from intact mice (No Cx), and from mice treated with castration alone (Cx) or castration + dutasteride (Cx+Dut) at early time points (d3-21, while still on therapy, indicated by double-headed arrows), or at castration-resistant re-growth (defined as >750 mm^3^). P values computed from Welch’s two sample t test (p<0.05 were considered significant). Single stars indicate a statistically significant difference in DHT levels between Cx vs. Cx+Dut treated samples at d3-21 of treatment. Double stars indicate a significant difference in DHT levels between Cx vs. Cx+Dut treated samples even after castration-recurrent re-growth. No other comparisons between Cx vs. Cx + Dut treated groups were significant. Expression of full length (FL) AR and the AR variant 7 (ARV7) truncated splice variant was measured in LuCaP35 (**C**) and LuCaP96 (**D**) at the time of tumor re-growth to 750 mm^3^. Transcript expression was measured by qRT-PCR and normalized to expression of the housekeeping gene RPL13A within each sample to yield the delta cycle threshold (dCt). The relative difference in expression between the indicated treatment groups was calculated using the delta dCt method (fold change = 2∧ddCt). P values computed from Welch’s two sample t test (p<0.05 were considered significant).

To determine whether the differential impact of SRD5A inhibition on tumor growth reflected a difference in suppression of intratumoral androgens, we measured T and DHT levels in tumors resected after treatment. In both LuCaP35 and LuCaP96 tumor types, mean tumor DHT levels measured at 3–21 days (indicated by two-headed arrows in **Figure 4A and 4B**) were substantially lower in castration plus dutasteride vs. the castration alone groups, with values in LuCaP35 suppressed from 0.40±0.56 ng/gm to 0.02±0.04, p = 0.007 and in LuCaP96 from 0.10±0.08 ng/gm to 0.02±0.01, p = 0.005 (summarized in **Table S1**).

Interestingly, while T levels in LuCaP35 tumors remained suppressed in either treatment group (**Figure 4A**, black bars), DHT levels in LuCaP35 tumors recurring after combined therapy (average 211 days after discontinuation of dutasteride) were similar to tumors recurring after castration alone (0.54±0.46 ng/gm and 0.61±0.63 ng/gm, respectively; gray bars). In contrast, DHT levels in LuCaP 96 tumors recurring after combined therapy (average 96 days after discontinuation of dutasteride) remained suppressed months after cessation of dutasteride (**Figure 4B**, 0.04±0.04 ng/gm compared to 0.53±0.46 ng/gm for castration alone), whereas T levels showed significant reconstitution after either castration alone or after combined therapy (1.34±0.89 ng/gm and 0.79±0.71 ng/gm, respectively, and **Table S1**). Of note, the half-life of dutasteride in mice is less than 2 days, [33] precluding a residual effect of SRD5A inhibition on steroid concentrations in 96 versus 35 tumors at the time of resection. Tumor androgen levels in LuCaP35 tumors at the time of re-growth were not associated with tumor size at enrollment (data not shown).

The tendency toward reconstitution of DHT levels in LuCaP35 and of T in LuCaP96 is consistent with the intrinsic variation in androgen metabolic programs favoring DHT vs. T identified in the untreated tumors (**Figure 1E**). Further, the relative expression of steroidogenic genes in LuCaP35 vs. LuCaP96 tumors recurring after castration vs. castration plus dutasteride was very similar to the expression patterns observed in untreated tumors (**Figure S2**). Collectively, these data show that dutasteride effectively inhibited DHT production in both tumor types, and that LuCaP35 tumor growth is susceptible to the enhanced pre-receptor suppression of DHT achieved with the combination of castration plus SRD5A inhibition, whereas LuCaP96 is not.

### Systemic androgen suppression plus SRD5A inhibition is associated with tumor type-specific differences in the induction of full length AR and AR splice variants

Castration resistant prostate tumors are frequently characterized by increased expression of full-length AR (AR^FL^), and as recently reported, by increased expression of AR^sv^ that lack the LBD and consequently confer constitutive ligand-independent AR activity. Differences in AR^FL^ and AR^sv^ levels may contribute to the variation in tumor growth responses following pre-receptor ligand suppression. Therefore, we evaluated the expression of AR^FL^ and the prevalent AR^sv^ identified in human CRPC samples, AR^V7^ (which encodes the same protein as AR^V3^) in LuCaP35 and LuCaP96 tumors recurring after ADT and dutasteride [13], [14], [16], [17], [34].

Compared to tumors resected from intact mice, LuCaP35 tumors recurring after castration or after castration plus dutasteride demonstrated a 2.0-fold increase in expression of AR^FL^, and no increase in AR^V7^ (**Figure 4C**, and **Table S3**). In contrast, while LuCaP96 tumors recurring after castration alone demonstrated a similar 1.9-fold increased expression of AR^FL^, they also showed a substantial 10-fold increase in the expression of AR^V7^ (**Figure 4D**). Moreover, LuCaP96 tumors recurring after castration plus dutasteride showed both a substantial 6.4-fold increase in AR^FL^ and a 7.8-fold increase in expression of AR^V7^ compared to untreated tumors. Decreased expression of androgen regulated genes such as PSA, TMPRSS2, NKX3.1, and FKBP5 was observed in tumors resected at d3-21 after initiation of treatment (data not shown). However, significant differences were generally not observed in recurrent tumors resected at late time points, consistent with the increase in AR expression (**Table S3**) and reconstitution of tumor androgen levels observed in these samples. Interestingly, expression of FKBP5 remained quite low in recurrent LuCap96 tumors despite the marked increase in AR^V7^, suggesting AR^V7^ does not necessarily completely recapitulate the transcriptional activity of full length AR.

Despite the increased expression of AR^V7^ in LuCaP96, we did not observe a parallel increase in the absolute expression of genes previously suggested to constitute the variant transcriptome (e.g. AKT, UBE2C, CDC20, CDK1, CYCLINA2; data not shown). (13–16) However the induction of AR^V7^ in LuCap96 was associated with a significant shift in the correlation of its expression with AKT. Whereas the expression of AR^V7^ and AKT was correlated in LuCaP35 tumors under all treatment conditions (Spearman r 0.7, 0.9, 0.6 in intact, castration alone and castration plus dutasteride groups, respectively, p<0.05 for all), expression of these genes only became correlated in LuCaP96 tumors when AR^V7^ was induced after treatment (Spearman r 0.2, 0.6, 0.8, p = 0.3, p = 0.002, p = 0.06, respectively, for intact, castration alone and castration plus dutasteride).

These data suggest that tumor-specific differences in the induction of full-length AR and AR^sv^ may account for the differential impact of pre-receptor androgen suppression on tumor-growth inhibition, with the marked rise in AR^V7^ observed in LuCaP96 rendering it correspondingly less sensitive to ligand suppression by dutasteride. Of note, consistent expression of the AR^v567^ variant also reported in human CRPC tissues was not detected in these xenografts (data not shown) [14], [16].

### Human CRPC metastases demonstrate patient-specific associations between tumor androgen levels and the expression of full length AR and AR splice variants

The studies of LuCaP35 and LuCaP96 xenografts support the existence of at least two subtypes of CRPC. One type is reliant on androgen biosynthesis and intratumoral maintenance of the potent AR ligand DHT, with modest upregulation of ligand-activated AR^FL^ (as represented by LuCaP35), whereas another is characterized by robust induction of AR^sv^, rather than metabolic adaptations directed at increased DHT synthesis (as represented by LuCaP96). To determine whether these observations are relevant in context of *in vivo* human prostate cancer, we compared relative levels of T, DHT and AR expression in tumor metastases directly obtained from men with CRPC. Given the 5–10 fold higher potency of DHT for engaging and activating AR relative to T, [26] we estimated an ‘absolute androgenicity index’ to more accurately model the total steroid ligand contribution present in each tumor, calculated as the sum of (5xDHT) + (1xT). Since well-qualified antibodies specific to AR^sv^ are not available, AR immunostaining was performed using antibodies recognizing the N-terminus (ARN+) or C-terminus (ARC+) of the AR protein to identify tumors expressing predominantly AR^FL^ (as assessed by both N-terminal and C-terminal immunoreactivity), and those with C-terminal truncated AR^sv^ (as assessed by N-terminal immunoreactivity but loss of C-terminal staining) [31].

**Figure 5:**
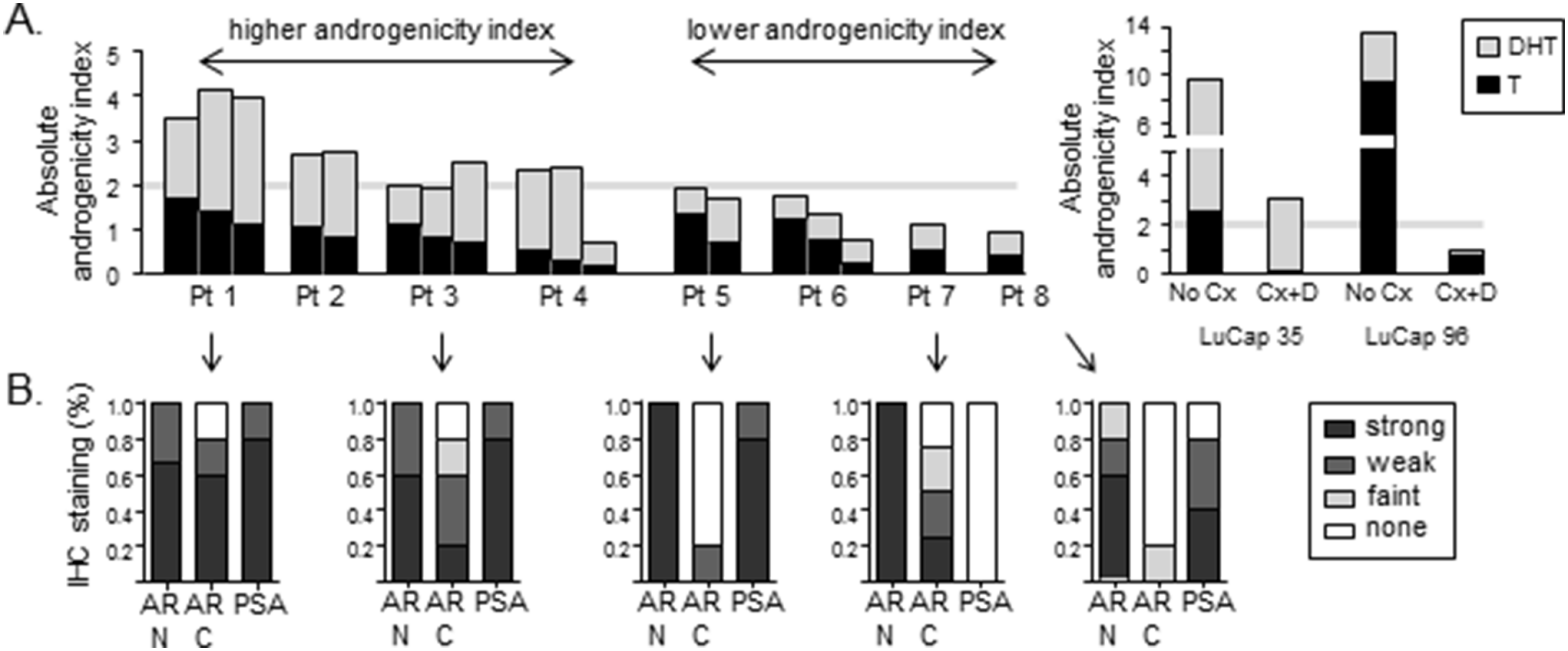
Androgen levels and expression of AR isoforms in castration resistant prostate tumor metastases. (**A**) Androgen levels were measured by mass spectrometry in 1–3 soft tissue metastases obtained from each of 8 patients via rapid autopsy. The graph depicts the absolute androgenicity index in each tumor, calculated using a 5∶1 ratio for the relative potency of DHT to T (e.g (5×DHT)+(1×T)). The portion of the total androgenicity contributed by T or DHT is represented by the stacked black and gray bars, respectively. The gray line represents a hypothetical cut point in the androgenicity index between tumors with relatively higher vs. relatively lower tissue androgenicity. Data for T and DHT in LuCaP35 and LuCaP96 tumors from intact mice (No Cx) or recurring after combined hormonal therapy (castration + dutasteride, Cx+D) is presented for comparison. (**B**) IHC staining scores for AR and PSA expression in 4–5 separate tumor metastases from five of the patients presented in panel A (as indicated by arrows). The % of each patient’s tumors demonstrating no, faint, weak or strong staining for the indicated antibody is presented. AR stains were separately performed using either N or C terminal antibodies to identify N+ but C– tumors consistent with the presence of C terminal truncated AR variants.

Notably, patients could be grouped into subsets whose metastases had a relatively high vs. relatively low androgenicity index, similar to that observed for treatment-recurrent LuCaP35 and LuCaP96 tumors (above or below an arbitrary cutpoint derived from visual inspection; **Figure 5A** **)**. Moreover, patients 1 and 3 whose metastases were characterized by a higher androgenicity index with a relatively larger contribution of DHT (similar to castration recurrent LuCaP35) demonstrated primarily ARN+ and ARC+ staining, consistent with detection of AR^FL^, and were consistently PSA positive (**Figure 5B**). In contrast, patients 5, 7, and 8 whose metastases were characterized by a lower androgenicity index with a relatively lower contribution of DHT (and more similar to castration recurrent LuCaP96), demonstrated loss of AR C-terminal staining and more variable PSA staining, consistent with the expression of truncated AR^sv^.

## Discussion

Defining factors that influence the sensitivity of prostate cancers to therapeutics targeting the AR-axis is critical for predicting treatment efficacy and identifying resistance mechanisms to prioritize future research efforts. Using two distinct models of castration sensitive advanced prostate cancer, we identified tumor-specific differences in intratumoral androgen levels and expression of AR^FL^ and AR^SV^ which associate with response and resistance to pre-receptor suppression of AR ligands. Evaluating these findings in clinical specimens acquired directly from men with CRPC indicate that observations made in the xenograft models are relevant to the spectrum of disease encountered in patients with advanced prostate cancer.

We hypothesized that the impact of SRD5A inhibition on tumor growth would associate with intra-tumoral androgen levels and/or expression of steroid biosynthetic and catabolizing enzymes suggestive of a relative dependence on DHT-mediated growth. In comparing tumor lines with an apparently similar clinical sensitivity to castration, we find a marked difference in the efficacy of combining SRD5A inhibition with systemic androgen suppression. We find that sensitivity in one model, LuCaP35, associates with evidence of DHT-dependent growth, whereas resistance in a second model, LuCaP96, associates with evidence of DHT-independent growth and the induction of AR^FL^ and ligand independent AR^SV^, such that these latter tumors are resistant to SRD5A-targeted ligand inhibition. While SRD5A inhibitors alone are unlikely to have significant clinical efficacy in advanced CRPC tumors, the expression of steroidogenic enzymes, AR^FL^ and AR^SV^ may serve as predictors of efficacy for these agents in prostate cancer prevention, or as a component of ADT in certain patients with advanced disease.

Factors responsible for the preferential induction of steroidogenesis versus induction of AR^FL^ or AR^SV^ expression in LuCaP35 and LuCaP96, respectively, are unknown. The robust expression of AR^V7^ after castration alone in LuCaP96 may reflect the 4 fold lower DHT levels achieved by castration alone in this xenograft (0.10±0.08 ng/gm) compared to LuCaP35 (0.40±0.56 ng/gm), as emerging data suggest that induction of AR variants reflects a dynamic response to ligand inhibition. [35] However, AR^V7^ is not markedly induced by suppression of DHT to 0.02±0.04 ng/gm in LuCaP35 tumors treated with castration plus dutasteride, suggesting tumors may also possess intrinsic differences in the propensity for generation of AR^SV^. Our data demonstrating significant levels of intratumoral T in LuCaP96 are in contrast to the study of Chang et al, which suggested that the route to intratumoral DHT synthesis can bypass T. Our findings indicate that this is not the case in every instance, and emphasize the range of tumor resistance mechanisms likely to be encountered in CRPC tumors [36].

The impact of DHT suppression on tumor growth inhibition in LuCaP35 was influenced by tumor size at initiation of therapy, with smaller tumors demonstrating a more profound suppression of tumor growth which also appeared to be durable after discontinuation of therapy, but larger tumors demonstrating suppression of tumor growth only while actively receiving treatment. These data suggest that even in tumors that are sensitive to androgen suppression, the potential efficacy associated with ligand synthesis inhibitors may be more pronounced and/or more durable if initiated earlier at a lower volume of disease. These data also provide a hypothesis for results of the PCPT and REDUCE clinical trials using SRD5A inhibitors for prostate cancer chemoprevention. [21], [22] Specifically, small volume cancers existing at the start of treatment may have regressed or been inhibited from progressing by exposure to SRD5A inhibition, whereas larger tumors existing at the start of therapy may have been less sensitive. Alternatively, the presence of AR^SV^, as has been observed in a subset of hormone naïve primary prostate cancers, [13], [17] might render these tumors less responsive to ligand requirements and thus less responsive to SRD5A inhibition.

Notably, direct examination of human CRPC tumors showed that *in situ* metastases demonstrate a patient-specific range of tumor androgen levels and associated differences in AR expression similar to that observed in LuCaP35 and LuCaP96 xenografts. Specifically, human metastases characterized by higher tissue androgen index demonstrated evidence of full length AR expression (similar to LuCaP35), whereas metastases with a relatively lower tissue androgen index demonstrated loss of C terminal AR staining, consistent with expression of ligand independent AR variants (as observed in LuCaP96). These data suggest that our findings regarding response and resistance to treatment strategies targeting pre-receptor androgen pathway suppression in the LuCaP35 and LuCaP96 xenograft models are likely to be relevant to the clinical spectrum of disease encountered in patients with CRPC.

Importantly, the observations reported in the present study may be relevant to optimizing first-line treatment strategies with novel ligand-synthesis and AR targeted agents such as abiraterone and enzalutamide. While the clinical responses to abiraterone and enzalutamide have been impressive, not all patients respond, the duration of response has been variable, and mechanisms of resistance are not well understood. [23], [24] Our findings are consistent with prior data demonstrating induction of steroidogenesis and/or increased expression of AR^FL^ or AR^SV^ as potential mechanisms of resistance in tumors recurring after treatment with abiraterone. [30] Notably, the induction of AR^SV^, at a magnitude that may be sufficient to impede sensitivity to additional androgen ablation, can occur following castration alone, as observed in LuCaP96 tumors. Clearly, these represent prostate tumors for which agents acting through mechanisms independent of ligand binding have a higher likelihood of efficacy than agents targeting ligand synthesis or the LBD. Consistent with this hypothesis, recent data demonstrate that patients with detectable AR^SV^ in circulating tumor cells have a poor response to the LBD antagonist enzalutamide. [37] Conversely, patients with tumors resembling LuCaP35, which demonstrate robust expression of steroidogenic enzymes consistent with maintenance of DHT levels, but without significant induction of ligand-independent AR variants, may represent an enriched population of patients in whom ligand synthesis inhibitors are likely to have the highest degree of efficacy. This hypothesis is consistent with recent data associating AR and CYP17A staining in tumor samples of men with CRPC and response to the ligand synthesis inhibitor abiraterone [38].

An important limitation of this study is that only two xenograft models were characterized, and evaluation of further models will be required to establish the validity of AR^SV^ and steroidogenic enzyme expression as biomarkers for treatment success. Collectively, however, these studies suggest that intrinsic differences in basal steroidogenesis and expression of ligand-independent AR^SV^ may associate with response and resistance to pre-receptor suppression of AR ligands, and may directly underlie the variable clinical efficacy observed in clinical studies of abiraterone and enzalutamide in men with CRPC. Recent data demonstrating that enzalutamide, which targets the AR LBD, had decreased efficacy against tumors expressing LBD-deleted AR^SV^, suggests these tumors may require agents targeting the AR N terminus, and is a critical subject of ongoing research. [35], [37], [39] The utility of identifying the expression of steroidogenic enzymes, AR^FL^ and AR^SV^ as potential biomarkers of response and resistance requires assessment in ongoing clinical studies employing molecular evaluation of tumor tissue from men with CRPC.

## Supporting Information

Figure S1 **The Androgen Synthetic Pathway.** The schema depicts the enzymatic steps in the conversion of cholesterol to androgens. Steroid intermediates are given in the boxes. Steroidogenic enzymes mediating each step are given next to the arrow denoting the direction of the conversion. After being generated from cholesterol by CYP11A C21 steroids are converted to the C19 adrenal androgens DHEA by the sequential hydroxylase and lyase activity of CYP17A. DHEA is acted on by HSD3B to form androstenedione (AED), which in the classical pathway is then acted on by HSD17B3 (or AKR1C3) to form testosterone, which is converted to DHT via SRD5A. In the 5-alpha-Androstanedione pathway AED is converted first by SRD5A to 5α-Androstanedione and then by HSD17B3 (or AKR1C3) to DHT. In the backdoor pathway the progestin intermediates are acted on first by the activity of SRD5A and the reductive activity of AKR1C2 prior to the lyase activity of CYP17A to form androsterone (not shown). Androsterone is then acted on by HSD17B3 (or AKR1C3) and must undergo an oxidative step mediated by RL-HSD (or others) to generate DHT.
(TIF)

Figure S2 **Expression of steroidogenic genes in LuCap35 and LuCaP96 prostate cancer xenografts.** Relative expression of the indicated steroidogenic genes in LuCaP35 and LuCaP96 in tumors grown in intact mice **(A, replicated from** **Figure 1E** **for comparison)** and in tumors re-growing after castration **(B)** or castration+8 weeks dutasteride **(C)**. Transcript levels were measured by qRT-PCR and normalized to the housekeeping gene RPL13A within each sample to yield the delta cycle threshold (dCT). For each gene the relative difference in mean expression between LuCaP35 and LuCaP96 was calculated using the delta dCt method (fold change = 2∧ddCtT). Genes differentially expressed in LuCaP35 vs. LuCaP96 within one order of magnitude are indicated within the gray lines. Significant differences (by Welch’s t test; p<0.05) are indicated by black circles and colored triangles; white circles indicate genes that were not significantly different between LuCaP35 and LuCaP96. Upward red triangles indicate highly differentially expressed genes leading to increased T (AKR1C3) and increased DHT levels (SRD5A1, RLHSD, 17BHSD10). Downward green triangles indicate highly differentially expressed genes mediating DHT catabolism (AKR1C2, AKR1C1, UGT2B15).
(TIF)

Table S1 **Androgen levels in LuCaP35 and LuCaP96 tumors after pre-receptor androgen suppression with castration or castration + dutasteride.**
(TIF)

Table S2 **Fold difference in mean transcript expression of steroidogenic genes between LuCaP35 and LuCaP96 tumors grown in intact mice.**
(TIF)

Table S3 **Expression of AR and AR-regulated genes in LuCaP35 and LuCaP96 tumors after pre-receptor androgen suppression with castration or castration + dutasteride.**
(TIF)
