# Supplementary figures and images for: Prostate Cancer Characteristics Associated with Response to Pre-Receptor Targeting of the Androgen Axis
Source: PLoS One. 2014 Oct 30;9(10):e111545. doi: 10.1371/journal.pone.0111545 (PMC4214744; doi:10.1371/journal.pone.0111545)

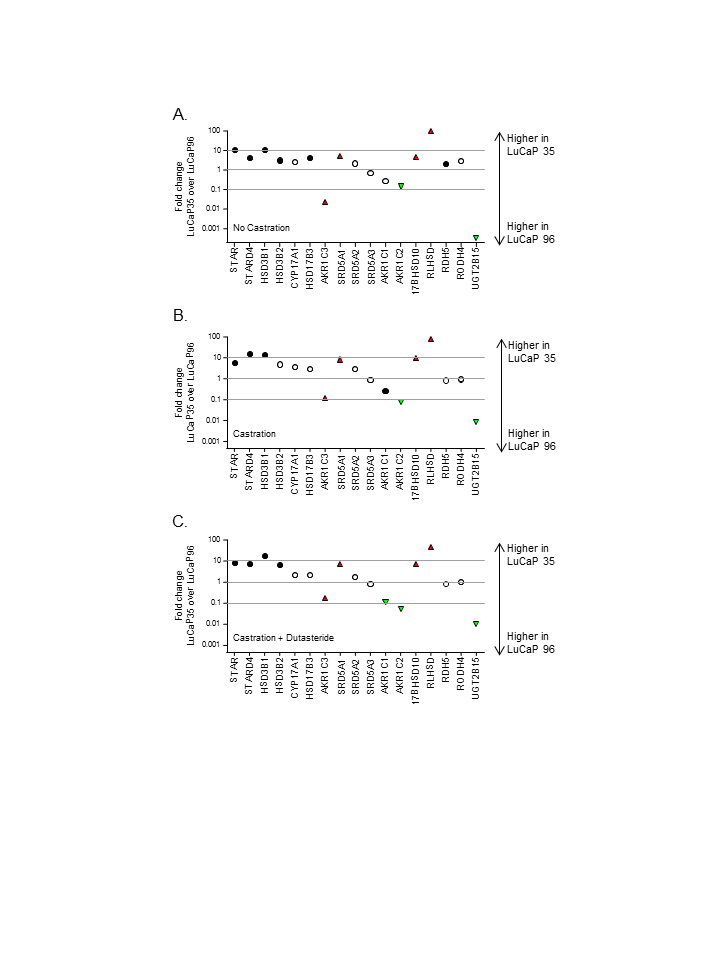

Supplement: Figure S2 — Expression of steroidogenic genes in LuCap35 and LuCaP96 prostate cancer xenografts. Relative expression of the indicated steroidogenic genes in LuCaP35 and LuCaP96 in tumors grown in intact mice (A, replicated from Figure 1E for comparison) and in tumors re-growing after castration (B) or castration+8 weeks dutasteride (C). Transcript levels were measured by qRT-PCR and normalized to the housekeeping gene RPL13A within each sample to yield the delta cycle threshold (dCT). For each gene the relative difference in mean expression between LuCaP35 and LuCaP96 was calculated using the delta dCt method (fold change = 2∧ddCtT). Genes differentially expressed in LuCaP35 vs. LuCaP96 within one order of magnitude are indicated within the gray lines. Significant differences (by Welch’s t test; p<0.05) are indicated by black circles and colored triangles; white circles indicate genes that were not significantly different between LuCaP35 and LuCaP96. Upward red triangles indicate highly differentially expressed genes leading to increased T (AKR1C3) and increased DHT levels (SRD5A1, RLHSD, 17BHSD10). Downward green triangles indicate highly differentially expressed genes mediating DHT catabolism (AKR1C2, AKR1C1, UGT2B15). (TIF) [file pone.0111545.s002.tif]

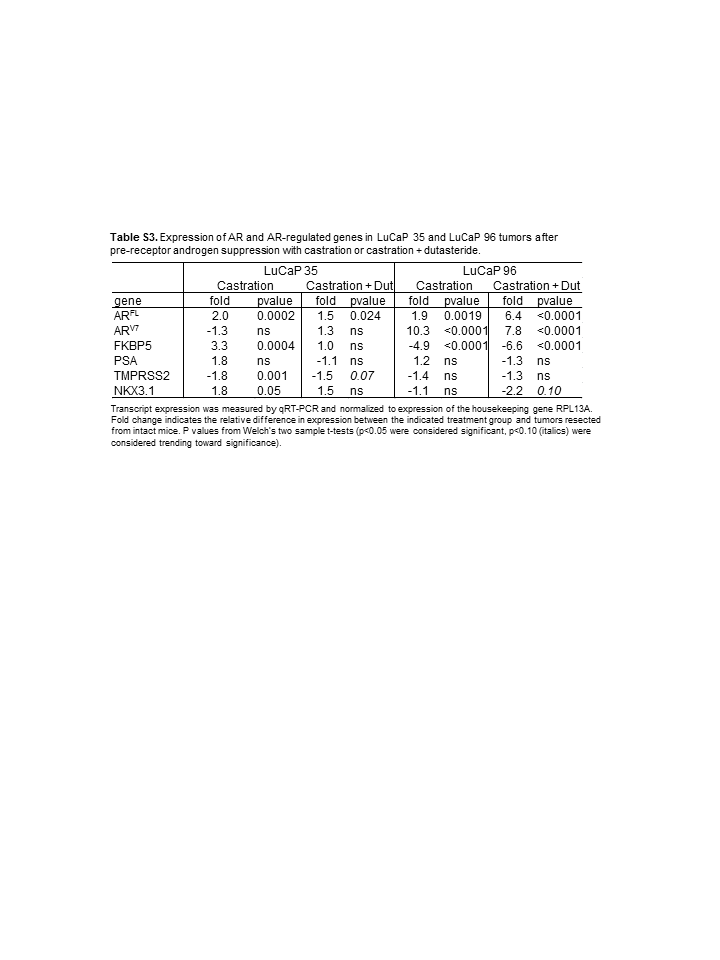

Supplement: Table S3 — Expression of AR and AR-regulated genes in LuCaP35 and LuCaP96 tumors after pre-receptor androgen suppression with castration or castration + dutasteride. (TIF) [file pone.0111545.s005.tif]
